# Anemia Among Patients Undergoing Transcatheter Mitral Valve Repair: From the National Inpatient Sample in the United States

**DOI:** 10.7759/cureus.10074

**Published:** 2020-08-27

**Authors:** Bhaskar Bhardwaj, Poorna R Karuparthi, Rupak Desai, Hee Kong Fong, Kul Aggarwal

**Affiliations:** 1 Department of Internal Medicine, Division of Cardiovascular Medicine, University of Missouri-Columbia, Columbia, USA; 2 Department of Cardiology, Atlanta Veterans Affairs Medical Center, Decatur, USA; 3 Department of Cardiovascular Medicine, University of California Davis Medical Center, Sacramento, USA; 4 Department of Internal Medicine, Division of Cardiovascular Diseases, University of Missouri-Columbia, Columbia, USA

**Keywords:** transcatheter mitral valve repair, iron deficiency anemia (ida), valvular heart disease, chronic anemia, mitral regurgitation

## Abstract

Background

The prevalence and impact of anemia on the outcomes of transcatheter mitral valve repair (TMVr) have not been well-studied. Anemia is a commonly encountered comorbidity among patients with cardiovascular disorders and is frequently under-recognized. The study aimed to analyze the prevalence of anemia and its impact on post-TMVr in-hospital outcomes.

Methods

The National Inpatient Sample (NIS) was queried to identify all patients who underwent TMVr from 2011-2015 in the United States by utilizing suitable International Classification of Diseases, Ninth Revision (ICD-9) codes. The baseline characteristics and in-hospital outcomes were compared among patients with and without anemia.

Results

A total of 4,382 patients were identified. Out of these, 978 (22.3%) patients had baseline anemia. Anemic patients were noted to have a higher burden of co-morbidities, including chronic kidney disease, hypertension, and diabetes mellitus. The in-hospital mortality was higher but not statistically significant between anemic and non-anemic patients (3.6% vs 2.6%; odds ratio (OR): 1.44; confidence interval (CI): 0.85-2.46, p=0.179). The other adverse outcomes, including the length of stay, the requirement for blood transfusions, the incidence of post-implant acute kidney injury, hemodialysis, and the cost of hospitalization, were higher in anemic patients.

Conclusion

Anemia was present in one out of five patients undergoing TMVr in this nationally representative cohort. Baseline anemia showed numerically higher but not statistically significant in-hospital mortality and was associated with other in-hospital adverse outcomes. Further larger studies are needed to highlight the importance of anemia in the TMVr procedure.

## Introduction

Mitral regurgitation (MR) is the commonest valvular disorder in developed countries with degenerative MR being the most etiology [1-2]. Transcatheter mitral valve repair (TMVr) using MitraClip (Abbott Vascular, Santa Clara, California) has been established as a viable and effective option for MR patients with high or prohibitive surgical risk [3-7]. Anemia is a commonly encountered comorbidity in elderly patients with cardiovascular disorders [8-9]. Its association with poor outcomes in several cardiovascular disorders, cardiac surgeries, and percutaneous procedures is well-described [9-17]. Among the percutaneous valvular interventions, prior studies regarding anemia in patients undergoing transcatheter aortic valve replacement (TAVR) have shown higher mortality and increased requirement for blood transfusions [15-17]. Data regarding the impact of baseline anemia in patients with MR undergoing TMVr are limited. This study aimed at analyzing the prevalence of anemia and its impact on in-hospital outcomes among patients undergoing TMVr in a large nationally representative cohort in the US.

## Materials and methods

Data source

The study cohort of TMVr procedures among adult patients was derived from the National Inpatient Sample (NIS) from January 2011 to September 2015. NIS is designed by the Agency for Healthcare Research and Quality (AHRQ) under Healthcare Cost and Utilization Project (HCUP). It is the largest all-payer inpatient dataset in the US [18-19]. Before 2011, it comprised a stratified sample of 20% nonfederal US community hospitals and was called Nationwide Inpatient Sample [19]. Afterward, the NIS included data from all hospitals participating in the HCUP. The NIS dataset provides estimates of over 35 million nationwide hospitalizations annually after being weighted. Each hospitalization in the NIS contains de-identified data with one primary diagnosis and up to 24 secondary diagnoses for a particular hospitalization along with hospitalization details such as location/teaching status, bed size, and geographical region of hospitals. Healthcare resource consumption in terms of median length of stay (LOS) in days, hospital charges incurred per admission, and discharge status of patients are also integrated. This dataset reports diagnoses and procedures using the International Classification of Diseases, 9th revision, Clinical Modification (ICD-9 CM) codes.

Study population

**Figure 1:**
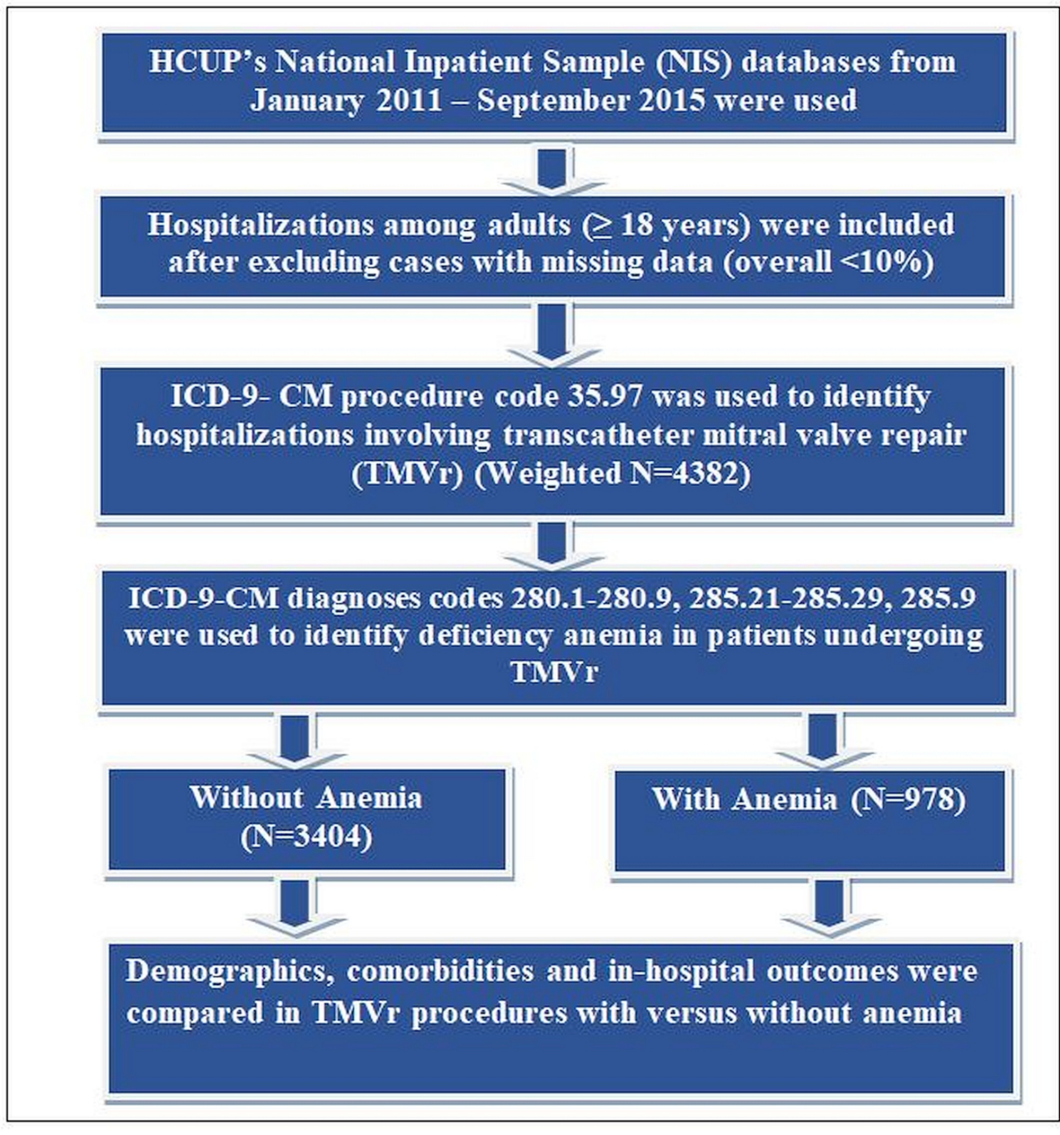
Cohort figure This figure shows the algorithm for study cohort selection.

TMVr-related admissions among the adult population were selected using ICD-9 CM procedure codes 35.97. The hospitalizations with missing data were excluded from the analysis (<10%). Baseline anemia was recognized by ICD-9 CM codes 280.1-280.9, 285.21- 285.29, 285.9 (Figure 1), and defined as a hemoglobin level < 12 g/dl in women and < 13 gm/dl in men to be defined as anemia as per the World Health Organization (WHO) definition [20]. These codes have been previously utilized to identify patients with anemia [21-22]. AHRQ comorbidity measures were utilized as predefined in the database. Table 1 shows, in detail, the ICD-9 CM/Clinical Classification Software (CCS) codes used to identify the comorbidities and complications employed to perform this analysis.

**Table 1:** ICD-9 CM & CCS codes used to identify comorbidities and complications ICD-9 CM & CCS codes used to identify comorbidities and complications. Abbreviations: ICD-9 = International Classification of Diseases, 9th revision, Clinical Modification (ICD-9 CM); CCS = Clinical Classification Software; TMVr = Transcatheter mitral valve repair; PVD = Peripheral vascular disease; IQR = Interquartile range; HMO = health maintenance organization, HCUP = healthcare cost and utilization project Categorical variables are presented as percentages (%) of weighted number of patients in each subgroup. Continuous variables are presented as mean values ± 1 standard deviation. (-) = not reported

Comorbidities/complications	ICD-9 CM/CCS codes
All anemias	ICD-9 280.0-280.9, 285.21-285.29, 285.9
Peripheral vascular disease	ICD-9 440.x, 441.x, 442.x, 443.1, 443.2x, 443.8x, 443.9, 444.x, 445.0, 447.1, 557.1, 557.9, 249.7x, 250.7x
Dyslipidemia	CCS 53
Smoking	ICD-9 305.1 V15.82
Hemodialysis status	ICD-9 V45.11
Thrombocytopenia	ICD-9 287.5, 287.30, 287.31, 287.33, 287.39, 287.49
Previous myocardial infarction	ICD-9 412
Previous percutaneous coronary intervention	ICD-9 V45.82
Previous coronary artery bypass grafting	ICD-9 V45.81
Atrial fibrillation	ICD-9 427.31
Mechanical circulatory support	ICD-9 procedure code 37.6x
Cardiac arrest	ICD-9 427.5, 427.1, 427.41, 427.42, 799.1, 99.60, 99.63
Cardiogenic shock	ICD-9 785.51
Postoperative myocardial infarction	CCS 100
Iatrogenic cardiac complications	ICD -9 997.1, 429.4
Requirement for permanent pacemaker	ICD-9 37.70 to 37.79, 37.80 to 37.89, 00.50, 00.52, 00.53
Pericardial complications	ICD-9 423.0 423.3 423.9 37.0
Blood transfusion	ICD-9 99.00, 99.01, 99.02, 99.03, 99.04
Postoperative hypotension/shock	ICD-9 458.2 998.0
Perioperative stroke	ICD-9 997.02, 435.9, CCS 109
Intracranial hemorrhage	ICD-9 431
Acute kidney injury requiring dialysis	ICD-9 CM 584.9+ ICD-9 CM procedure 39.95
Post procedural respiratory failure	ICD-9 518.51, 518.52, 518.53, 518.81, 518.82, 518.84, 799.1
Venous thromboembolism	ICD-9 415.1x, 451.1x, 451.2, 451.8x, 451.9, 453.2, 453.4x, 453.8x, and 453.9
Post procedural infection	ICD-9 998.5, 998.51, 998.59, 999.3, 038.0, 995.91, 995.92
Gastrointestinal complications including hemorrhage	ICD-9 997.4 CCS 153

Study variables

The variables of interest regarding patient-level demographics included age, sex, race, elective/non-elective admission, primary payer type, median household income percentile as per patients’ zip code, hospital-level characteristics, including hospital bed size, location/teaching status, and region of hospitals. The analysis also assessed the prevalence of various pre-existing comorbidities between anemic and non-anemic groups. Comorbidities and in-hospital complications (in-hospital cardiac arrest, cardiogenic shock, postoperative myocardial infarction, iatrogenic cardiac complications, the requirement of a pacemaker, pericardial complications, postoperative hypotension, shock, perioperative stroke, acute kidney injury (AKI) requiring dialysis, postoperative respiratory failure, venous thromboembolism, and postoperative infection) were identified by using ICD-9 CM or CCS codes as mentioned in Table 1. The primary endpoint for the study was all-cause in-hospital mortality in TMVr. Secondary endpoints were in-hospital complications, the median length of stay (LOS), blood transfusion requirement, hospitalization charges incurred per admission, and discharge disposition. A multivariable analysis was performed to evaluate the odds of in-hospital outcomes in patients undergoing TMVr. The details of the covariates used for the multivariable analysis can be found in Table 2. Moreover, an additional analysis was done with fewer variables in the multivariable model (Table 2). Pearson’s chi-square test and the Mann-Whitney U test were performed to compare the categorical and continuous variables between the two groups. Results were expressed as numbers or percentages and median with interquartile range, respectively. Significant independent predictors on univariate analysis were incorporated into a multivariable regression analysis to evaluate the influence of anemia on in-hospital outcomes among patients undergoing TMVr. The outcomes were specified in terms of adjusted odds ratio (aOR) with a 95% confidence interval (95%CI). Linear regression analyses were also accomplished to evaluate resource usage indicative by the prolonged length of stay (days) and higher hospital charges in TMVr performed in patients with anemia. Multivariable analysis was performed adjusting for age, sex, race, type of admission (elective/non-elective), median household income, primary payer type, and baseline comorbidities considered clinically relevant (Table 2).

**Table 2:** Variables utilized in the multivariable model List of variables utilized for the multivariable models

List of variables utilized for the 1st multivariable model	List of variables for the 2^nd^ restricted multivariable model
Age, Sex, Race, Type of admission (elective/non-elective), Median household income, Payer status, Hospital bed size, and location/teaching status, Hypertension, Diabetes, Dyslipidemia, Obesity, Smoking, Rheumatoid arthritis/collagen vascular disease, Congestive heart failure, Chronic obstructive pulmonary disease, Coagulopathy, Chronic kidney disease, End stage renal disease, Hemodialysis status, Liver disorder, Previous myocardial infarction/revascularization, Mechanical circulatory support, Pacemaker status, Thrombocytopenia.	Age, Sex, Race, Type of admission (elective/non-elective), Hypertension, Diabetes, Dyslipidemia, Obesity, Smoking, Congestive heart failure, Chronic obstructive pulmonary disease, Coagulopathy, Chronic kidney disease.

Statistical analysis

Pearson’s chi-square test and the Mann-Whitney U test were performed to compare the categorical and continuous variables between the two groups. Results were expressed as numbers or percentages and median with interquartile range, respectively. Significant independent predictors on univariate analysis were incorporated into a multivariable regression analysis to evaluate the influence of anemia on in-hospital outcomes among patients undergoing TMVr. The outcomes were specified in terms of adjusted odds ratio (aOR) with a 95% confidence interval (95% CI). Linear regression analyses were also accomplished to evaluate resource usage indicative by the prolonged length of stay (days) and higher hospital charges in TMVr performed in patients with anemia. Multivariable analysis was performed adjusting for age, sex, race, type of admission (elective/non-elective), median household income, primary payer type, and baseline comorbidities considered clinically relevant (Table 2).

A two-tailed p <0.05 was considered statistically significant. The Statistical Package for the Social Sciences (SPSS) v24 (IBM Corp, Armonk, NY) and complex sample modules were utilized to complete statistical analyses using defined strata/cluster designs. The methodological strategy utilized in the analysis met the recommended standards [23].

## Results

**Figure 2:**
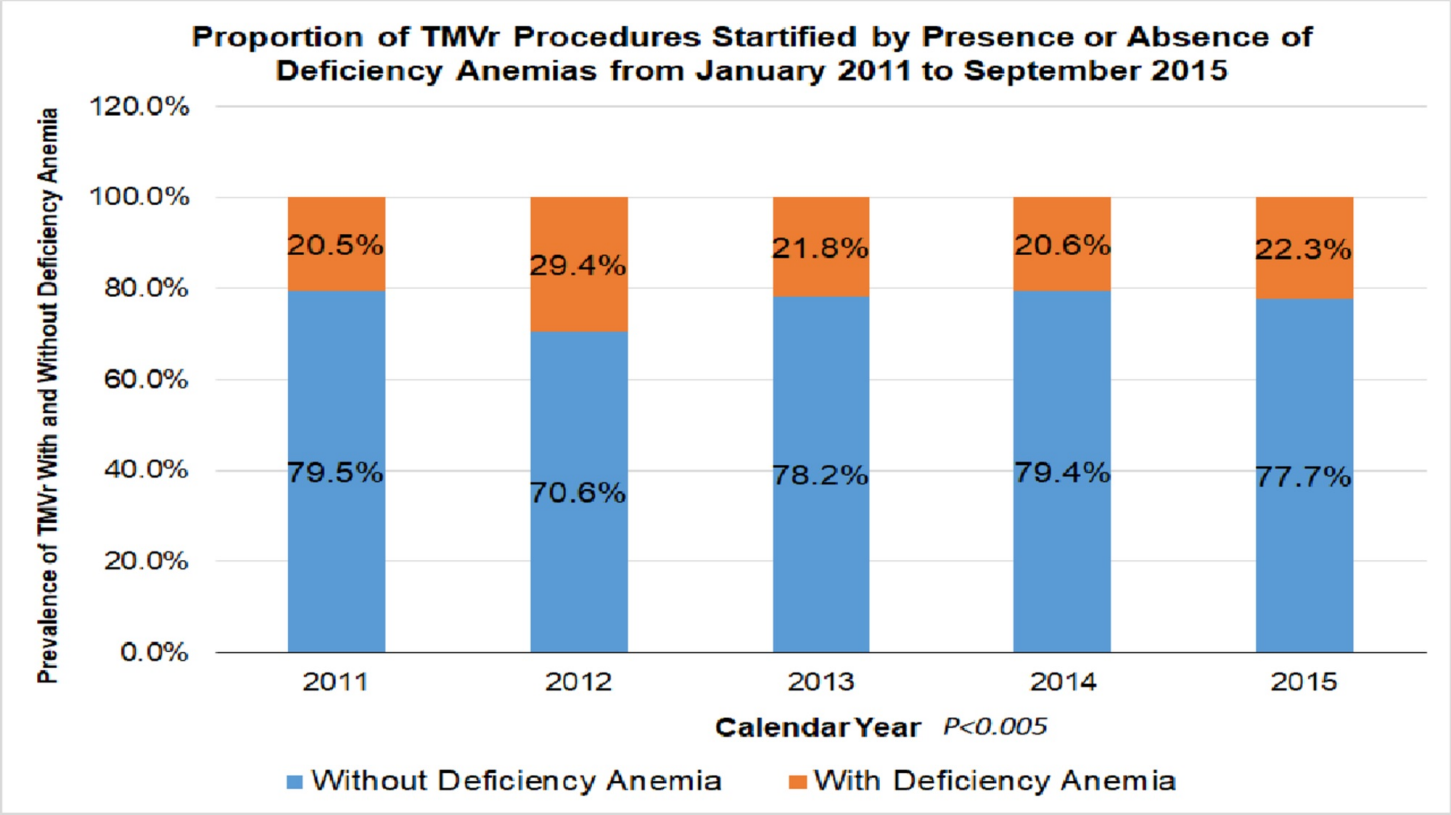
Proportion of anemic patients undergoing transcatheter mitral valve repair This figure demonstrates the proportion of anemic patients undergoing transcatheter mitral valve repair (in orange). The X-axis shows the percentage of patients with anemia (in orange) and those without (in blue). The Y-axis is the time in years.

From January 2011 to September 2015, a total of 4,382 patients underwent TMVr, out of which 978 (22.3%) had anemia. The proportion of anemic patients among all patients undergoing TMVr over the study duration did not change significantly (Figure 2). The baseline characteristics of the patients with or without anemia are shown in Table 3.

**Table 3:** Baseline characteristics of patients undergoing transcatheter mitral valve repair with and without anemia Categorical variables are presented as the percentage (%) of the weighted number of patients in each subgroup. Continuous variables are presented as mean values ± 1 standard deviation. TMVr = Transcatheter mitral valve repair, PVD = peripheral vascular disease, IQR = Interquartile range, HMO = Health maintenance organization, HCUP = Healthcare Cost and Utilization Project, (-) = not reported

Variable		With Anemia (N=978)	Without Anemia (N=3,404)	Overall (N=4,382)	P-value	
Age (years) at admission						
Median (IQR)		78 (66-84)	77 (67-84)	77 (67-84)	0.373	
18-44		2.0%	2.9%	2.7%	0.003	
45-64		21.0%	16.5%	17.5%		
≥65		77.0%	80.6%	79.8%		
Sex					0.086	
Male		54.5%	57.6%	56.9%		
Female		45.5%	42.4%	43.1%		
Race					<0.001	
White		72.1%	79.4%	77.8%		
African American		8.2%	5.8%	6.4%		
Hispanic		10.0%	8.0%	8.5%		
Asian or Pacific Islander		2.0%	2.6%	2.5%		
Native American		-	-	-		
Other		6.6%	4.1%	4.7%		
Primary Expected Payer					<0.001	
Medicare		79.6%	79.2%	79.3%		
Medicaid		6.1%	3.7%	4.2%		
Private including HMO		9.2%	15.1%	13%		
Self – Pay		2.0%	0.7%	1.0%		
No-charge		-	-	-		
Other		2.5%	1.2%	1.5%		
Median Household Income Quartile for Patients’ Zipcode					0.008	
0-25^th^		21.5%	22.5%	22.3%		
26-50^th^		22.3%	22.4%	22.4%		
51-75^th^		31.1%	26.1%	27.2%		
76-100^th^		25.1%	29.0%	28.2%		
Type of Admission					<0.001	
Non-elective		34.6%	23.9%	26.3%		
Elective		65.4%	76.1%	73.7%		
Bed size of Hospital					<0.001	
Small		7.8%	2.9%	4.0%		
Medium		16.3%	16.2%	16.2%		
Large		75.9%	80.9%	79.8%		
Region of Hospital					0.823	
Northeast		19.3	20.0	19.8		
Midwest		19.4%	18.5%	18.7%		
South		37.1%	36.3%	36.5%		
West		24.2%	25.2%	25.0%		
Location/Teaching Status of Hospital					0.01	
Rural		-	0.4%	0.3%		
Urban - non teaching		14.2%	11.5%	12.1%		
Urban - teaching		85.8%	88.0%	87.5%		
	P-values < 0.05 indicates statistical significance. ^*^Counts<11 are not reported (-) as per privacy guidelines by HCUP.					

There were a higher number of females in TMVr patients with vs without anemia (45.5% vs 42.4%, p<0.001). Among anemic patients undergoing TMVr, there was a higher proportion of African Americans (8.2% vs 5.8%, p<0.001) and Hispanic patients (10.0 vs 8.0%, p<0.001). There were more non-elective cases in patients with anemia (34.6% vs 23.9 %, p<0.001). Patients with anemia had a higher burden of co-morbidities with more patients having complicated diabetes, hypertension, chronic kidney disease (CKD), dialysis dependency, coagulopathy, thrombocytopenia, and peripheral vascular disease as seen in Table 4. Both groups had an equal likelihood of having rheumatoid arthritis, congestive heart failure (CHF), chronic pulmonary disease, prior percutaneous coronary interventions (PCI), prior coronary artery bypass, liver disease, and obesity. Mechanical circulatory support use was not different between both patient groups (Table 4).

**Table 4:** Baseline comorbidities in patients undergoing transcatheter mitral valve repair with and without baseline anemia

Comorbidities	With Anemia (N=978)	Without Anemia (N=3,404)	Overall (N=4,382)	P-value	
Rheumatoid arthritis/collagen vascular diseases	4.6%	5.5%	5.3%	0.244	
Congestive heart failure	1.5%	1.9%	1.8%	0.473	
Chronic pulmonary disease	26.0%	26.6%	26.5%	0.674	
Coagulopathy	19.9%	12.9%	14.4%	<0.001	
Diabetes, uncomplicated	22.5%	20.9%	21.2%	0.279	
Diabetes with chronic complications	8.1%	2.6%	3.9%	<0.001	
Hypertension	74.9%	70.6%	71.6%	0.009	
Dyslipidemia	56.6%	53.4%	54.1%	0.076	
Smoking	27.6%	31.4%	30.6%	0.022	
Previous myocardial infarction	16.3%	13.7%	14.3%	0.038	
Previous PCI	14.3%	15.5%	15.2%	0.359	
Previous CABG	22.8%	24.6%	24.2%	0.258	
Atrial fibrillation	55.1%	58.3%	57.6%	0.077	
Chronic kidney disease	47.3%	30.7%	34.4%	<0.001	
End stage renal disease	6.6%	2.3%	3.3%	<0.001	
Hemodialysis status	3.1%	0.7%	1.3%	<0.001	
Mechanical circulatory support device	3.7%	3.2%	3.3%	0.461	
Pacemaker status	13.2%	11.6%	12.0%	0.185	
Valvular heart disease	2.0%	2.0%	2.0%	0.972	
Thrombocytopenia	18.4%	10.4%	12.2%	<0.001	
Liver disease	2.0%	2.3%	2.3%	0.609	
Obesity	7.2%	8.8%	8.4%	0.101	
Peripheral vascular disorders	14.4%	10.4%	11.3%	<0.001	
Pulmonary circulation disorders	2.6%	0.9%	1.3%	<0.001	
P-values < 0.05 indicates statistical significance.					

The primary outcome of in-hospital mortality was higher in patients with anemia as compared to those without anemia but did not reach statistical significance on an adjusted analysis (3.6% vs 2.6%; aOR:1.44;CI:0.85-2.46, p=0.179). However, in adjusted analysis for secondary outcomes, the risk of AKI (18% vs 10.5%; aOR:1.53; CI:1.21-1.93, p <0.001), AKI requiring dialysis (3.1% vs 1.0%; aOR: 3.24; CI:1.75-5.99, p<0.001), blood transfusions (20.3% vs 13.0%; aOR 1.33; CI:1.08-1.63. p=0.008), and pericardial complications (4.1% vs 2.1%; aOR: 1.89; CI:1.17-3.07, p=0.009) was significantly higher in patients with anemia as compared to those without anemia. Moreover, patients with anemia undergoing TMVr had longer LOS {5 days (3-11) vs 3 days (2-7), p <0.001} and higher hospital charges {$169,413 ($120,391-$247,857) vs $151,895 ($114,080-$228,462), p<0.001} (Table 3). Additionally, the anemic patients undergoing TMVr were less likely to have a routine discharge disposition as compared to non-anemic patients (48.4% vs 62.1%; aOR: 0.57; CI: 0.48-0.67, p<0.001) as seen in Table 5. There was no difference in the outcomes on restricting the number of adjusted variables in the multivariable model demonstrated in Table 6.

**Table 5:** In-hospital outcomes of transcatheter mitral valve repair with and without anemia P<0.05 indicates statistical significance. #Multivariable analysis was performed adjusting for age, sex, race, type of admission (elective/non-elective), median household income, payer status, hospital bed size and location/teaching status, hypertension, diabetes, dyslipidemia, obesity, smoking, rheumatoid arthritis/collagen vascular disease, congestive heart failure, chronic obstructive pulmonary disease, coagulopathy, chronic kidney disease, end-stage renal disease, hemodialysis status, liver disorder, previous myocardial infarction/revascularization, mechanical circulatory support, pacemaker status, and thrombocytopenia. Concordance-statistics (C-index) for multivariable analysis > 0.70 *Unstandardized beta coefficient value from linear regression

Outcomes	With Anemia (N=978)	Without Anemia (N=3,404)	Overall (N=4,382)	P-value	aOR (95%CI)^#^	Adjusted P-Value
All-cause in-hospital mortality	3.6%	2.6%	2.8%	0.109	1.44 (0.85-2.46)	0.179
In-hospital cardiac arrest	2.0%	2.0%	2.0%	0.983	1.25 (0.66-2.36)	0.495
Cardiogenic shock	6.7%	3.8%	4.5%	<0.001	1.48 (0.96-2.29)	0.079
Iatrogenic cardiac complications	4.1%	4.1%	4.1%	0.993	0.87 (0.58-1.31)	0.52
Pericardial complications	4.1%	2.1%	2.5%	<0.001	1.89 (1.17-3.07)	0.009
Blood transfusion	20.3%	13.0%	14.6%	<0.001	1.33 (1.08-1.63)	0.008
Acute kidney injury (no dialysis)	18.0%	10.5%	12.2%	<0.001	1.53 (1.21-1.93)	<0.001
Acute kidney injury requiring dialysis	3.1%	1.0%	1.5%	<0.001	3.24 [1.75-5.99]	<0.001
Postoperative respiratory failure	4.1%	4.4%	4.3%	0.67	0.85 (0.56-1.30)	0.457
Venous thromboembolism	2.6%	1.8%	1.9%	0.112	1.32 (0.67-2.57)	0.418
Postoperative infection	3.6%	3.1%	3.2%	0.439	1.25 (0.61-2.55)	0.543
Gastrointestinal complications	2.6%	3.1%	3.0%	0.391	0.73 (0.45-1.19)	0.205
Disposition of patient				<0.001		
Routine discharge	48.4%	62.1%	59.0%		0.57 (0.48-0.67)	<0.001
Transfer to other (SNF, ICF)	17.8%	12.0%	13.3%			
Home health care	28.7%	21.8%	23.3%			
Length of stay (days), Median (IQR)	5 (3-11)	3 (2-7)	4 (2-8)	<0.001	1.17^*^	<0.001
Hospital charges (USD), Median (IQR)	169,413 (120,391-247,857)	151,895 (114,080-228,462)	158,319 (114,605-231,960)	<0.001	1497.82*	0.752

**Table 6:** In-hospital outcomes of TMVR with vs. without deficiency anemias in a restricted multivariable model TMVr = transcatheter mitral valve repair, aOR = adjusted odds ratio, CI = confidence interval, AKI = acute kidney injury, SNF = skilled nursing facility, ICF = intermediate care facility, HCUP = Healthcare Cost and Utilization Project P <0.05 indicates statistical significance. Counts <11 are not reported as per privacy guidelines by HCUP. #Multivariable analysis was performed adjusting for age, sex, race, type of admission (elective/non-elective), hypertension, diabetes, dyslipidemia, obesity, smoking, congestive heart failure, chronic obstructive pulmonary disease, coagulopathy, chronic kidney disease. Concordance-statistics (C-index) for multivariable analysis > 0.70

	In-hospital Outcomes of TMVR With vs. Without Deficiency Anemias						
Outcomes		With Anemia (N=978)	Without Anemia (N=3,404)	Overall (N=4,382)	P	aOR (95%CI)^#^	Adjusted P
All-cause in-hospital mortality		3.6%	2.6%	2.8%	0.109	0.97 (0.62-1.51)	0.877
In-hospital cardiac arrest		2.0%	2.0%	2.0%	0.983	0.75 (0.44-1.29)	0.300
Cardiogenic shock		6.7%	3.8%	4.5%	<0.001	1.37 (0.97-1.92)	0.074
Iatrogenic cardiac complications		4.1%	4.1%	4.1%	0.993	1.04 (0.73-1.50)	0.817
Pericardial complications		4.1%	2.1%	2.5%	<0.001	2.17 (1.45-3.26)	<0.001
Blood loss requiring transfusion		20.3%	13.0%	14.6%	0.001	1.33 (1.09-1.62)	<0.001
Acute kidney injury requiring dialysis		3.1%	1.0%	1.5%	<0.001	2.03 (1.05-3.92)	0.034
Postoperative respiratory failure		4.1%	4.4%	4.3%	0.67	0.84 (0.57-1.23)	0.365
Venous thromboembolism		2.6%	1.8%	1.9%	0.112	1.17 (0.71-1.94)	0.530
Postoperative infection		3.6%	3.1%	3.2%	0.439	0.73 (0.47-1.15)	0.171
Gastrointestinal complications		2.6%	3.1%	3.0%	0.391	0.73 (0.47-1.16)	0.190

## Discussion

**Figure 3:**
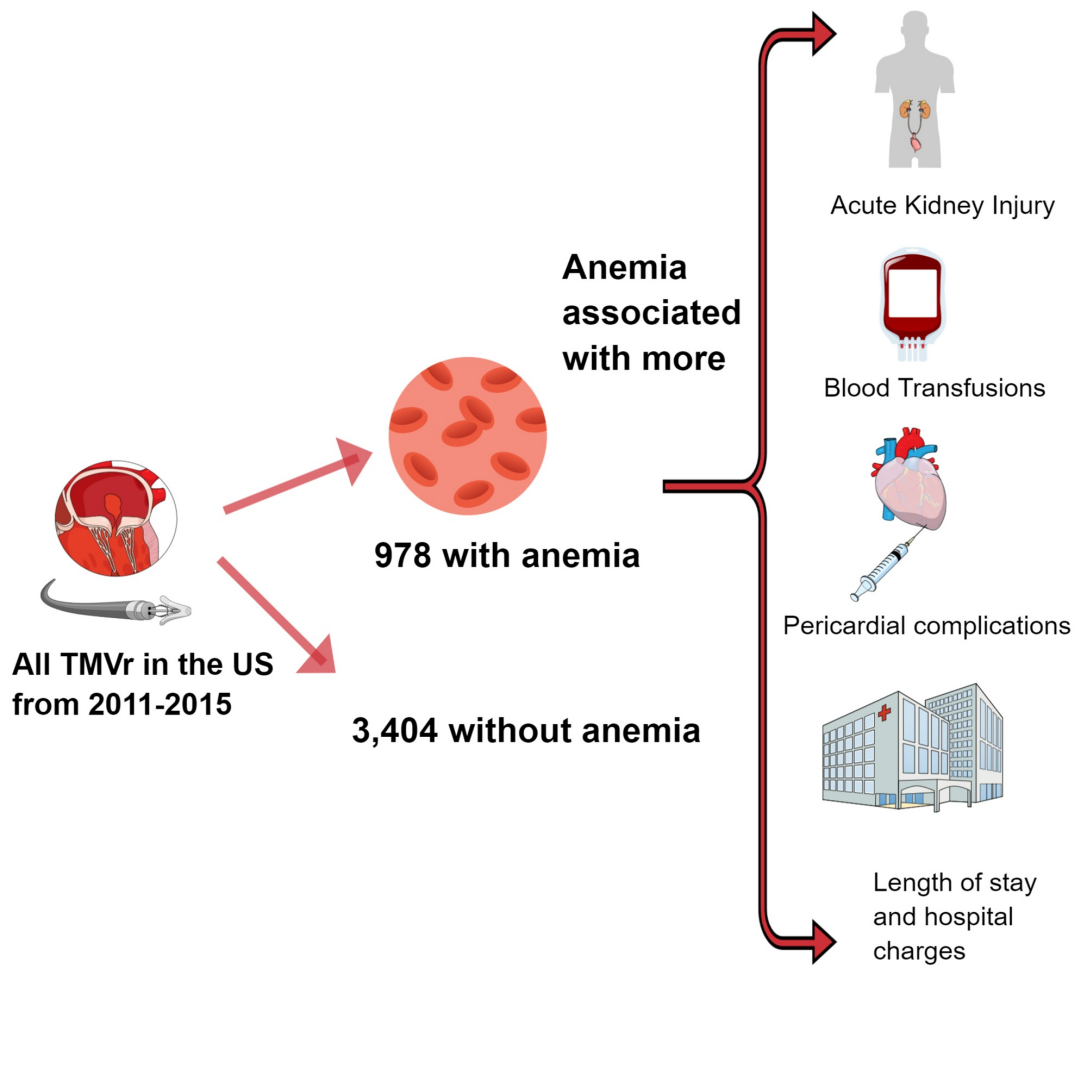
Summary figure This figure summarizes the key findings of the analysis. TMVr = Transcatheter mitral valve repair

The main findings from this study are: (1) Anemia is commonly encountered among patients undergoing TMVr, (2) Anemia in patients undergoing TMVr is associated with a higher risk of post-TMVr AKI, blood transfusions, pericardial complications, longer length of stay (LOS), and higher cost of hospitalization, and (3) Anemia in patients undergoing TMVr is associated with numerically higher in-hospital mortality as compared to patients without anemia (Figure 3).

Anemia is a frequently encountered comorbidity in the elderly population; as much as ~17% of patients above the age of 65 years have anemia [8]. In patients with CHF, anemia has a high prevalence and is associated with increased mortality, poor exercise capacity, and increased hospitalizations [24-26]. A meta-analysis summarizing the prior studies regarding the treatment of anemia in CHF had shown an improvement in mortality, exercise capacity, and CHF hospitalizations [26]. Among patients presenting for a PCI, anemia was associated with higher one-year mortality, prolonged LOS, and 30-day major adverse cardiovascular events [14]. Similarly, the prevalence of anemia in patients undergoing cardiac surgeries was 26-32%, and it was associated with poor outcomes [14,27]. Anemia is commonly encountered in patients undergoing TAVR with a prevalence reaching up to 57% and it is associated with similar 30-day mortality as patients without anemia [15,17]. However, the mid-term and long-term outcomes post-TAVR were found to be worse in anemic patients [15-17]. Prior single-center studies regarding anemia in patients undergoing TMVr reported a prevalence of up to 56% [11-12]. This difference in prevalence can be related to the ICD-9 codes-based identification of anemia in the current analysis and potentially overestimating coexisting anemia in smaller single-center studies previously [11-12].

The study by Hellhammer et al [11]. showed no difference in 30-day mortality and in-hospital complications among anemic and non-anemic patients undergoing TMVr, whereas Kaneko et al [12]. had demonstrated a lower survival in anemic patients at 6 months. Similar to the current study, both prior studies have shown a higher burden of comorbidities among anemic patients, including chronic kidney disease, diabetes mellitus, and hypertension [11-12]. The current analysis showed that patients with anemia had a higher prevalence of coagulopathy, thrombocytopenia, and peripheral vascular diseases. The in-hospital mortality among patients with anemia was higher (3.6% vs 2.4%) but did not reach a statistical significance on the adjusted analysis. The overall reported in-hospital mortality of ~ 2.8% in this analysis is comparable to real-world data from the Society of Thoracic Surgeons-Transcatheter Valve Therapy (STS-TVT) registry [7]. However, the data regarding the prevalence of anemia and its impact on outcomes among patients undergoing TMVr has not been reported in prior large analysis or clinical trials.

This study has some important clinical implications. It highlights anemia as prevalent comorbidity in patients undergoing TMVr and its association with post-procedure adverse events. Nonetheless, the patients with anemia undergoing TMVr remain at risk for developing AKI and requiring blood transfusions and dialysis, likely representing a higher proportion of a vulnerable population. The higher incidence of anemia and AKI has been described previously among hospitalized patients with acute myocardial infarction (MI) and was similarly seen in the current study [28]. The patients with anemia remain at risk for developing AKI even if the anemia develops during the hospitalization [29]. In the current era of expanding the use of TMVr in patients with functional mitral regurgitation, closer attention to the early identification and workup of underlying anemia is imperative [30]. Additionally, anemia can be considered a part of pre-procedure risk stratification tools for patients planned for a TMVr.

Limitations

This study has some notable limitations. First, the NIS does not collect the details of laboratory values; thus it was not possible to analyze the study cohort based on the severity/chronicity of anemia or the type/cause of anemia reliably. Second, the NIS relies on ICD-9 codes from the discharge documentation to extract details about the hospitalization, which is subject to administrative coding errors or under-coding. Moreover, the details of procedure or baseline/post-procedure echocardiographic data, information regarding access site bleeding, device-related adverse events, number of devices used, post-implant MR, and conversion to surgery are not available in the NIS, hence, could not be included in the analysis. Due to the limitations of the dataset, it is difficult to ascertain the timing and sequence of the events, e.g. day of hospitalization for in-hospital events or total units of blood transfusions given. Additionally, this analysis only focused on the in-hospital outcomes, and longitudinal data about long-term mortality or CHF hospitalizations are not available.

## Conclusions

In conclusion, this study describes the prevalence and in-hospital outcomes of TMVr in patients with anemia in a nationally representative cohort. Every fifth patient undergoing TMVr had underlying anemia in this administrative codes-based dataset. Anemic patients undergoing TMVr are associated with a higher risk for AKI and a higher requirement for blood transfusions and have longer LOS and higher costs of hospitalization, and numerically higher in-hospital mortality. Larger studies linking the severity and reversibility of anemia to outcomes are needed in the future with a focus on early identification.
